# How to Prevent Arrhythmias Following Acute Coronary Syndrome

**DOI:** 10.1002/clc.70086

**Published:** 2025-01-18

**Authors:** Naoya Kataoka, Teruhiko Imamura

**Affiliations:** ^1^ Second Department of Internal Medicine University of Toyama Toyama Japan

**Keywords:** atrial fibrillation, heart failure, ventricular arrhythmia

To Editor,

Ventricular arrhythmias (VAs) following acute coronary syndrome (ACS) are strongly associated with hemodynamic instability and increased mortality, underscoring the importance of accurate prediction for implementing prophylactic strategies. Giubertoni and colleagues demonstrated that the PRAISE score effectively identifies high‐risk patients for atrial fibrillation (AF) or VAs during hospitalization for ACS [1]. Nevertheless, several points warrant further consideration.

The authors employed clinical parameters required for calculating the PRAISE score [1], a tool originally developed using machine learning to predict 1‐year adverse cardiovascular and bleeding events following ACS [2]. However, additional potential predictors are known to influence arrhythmogenesis. For example, hyperuricemia and chronic obstructive pulmonary disease have been implicated in the development of AF, while specific electrocardiographic and echocardiographic parameters are associated with ischemia‐induced ventricular tachycardia [3, 4, 5]. Incorporating these established risk factors into a revised risk score may enhance its clinical utility.

Another critical consideration involves the hazard ratios of individual variables. Identifying modifiable risk factors provides actionable therapeutic targets to mitigate the incidence of AF and VAs post‐ACS. For instance, anemia emerged as a significant predictor in the original PRAISE cohort, alongside age and left ventricular ejection fraction [2]. Notably, anemia is widely recognized as a contributor to the pathogenesis of AF and may represent a practical focus for intervention.

The clinical implications of these findings remain ambiguous [1]. Cardiac reverse remodeling often occurs within approximately 30 days following ACS. During this period, the use of wearable cardioverter‐defibrillators may be appropriate, whereas implantable cardioverter‐defibrillators are typically not recommended. A pertinent question arises: how can referencing the PRAISE score inform strategies to improve mid‐ and long‐term clinical outcomes following ACS?

## Ethics Approval

The authors have nothing to report.

## Consent

The authors have nothing to report.

## Conflicts of Interest

The authors declare no conflicts of interest.

## Data Availability

The authors have nothing to report.
